# Supplementary material for: Manipulating fractional Shapiro steps in twisted cuprate Josephson junctions
Source: Natl Sci Rev. 2025 Dec 15;13(4):nwaf569. doi: 10.1093/nsr/nwaf569 (PMC12917811; doi:10.1093/nsr/nwaf569)
Supplement: nwaf569_Supplemental_File [file nwaf569_supplemental_file.docx]

**Supplementary Materials:**

**Manipulating fractional Shapiro steps in twisted cuprate Josephson junctions**

Yuying Zhu^1,4, †,^ *, Heng Wang^2, 3, †^, Ding Zhang^1, 3, 4,^ *, and Qi-Kun Xue^1, 2, 3,^ *

^1^Beijing Academy of Quantum Information Sciences, Beijing 100193, China

^2^ State Key Laboratory of Quantum Functional Materials, Department of Physics, and Guangdong Basic Research Center of Excellence for Quantum Science, Southern University of Science and Technology, Shenzhen 518055, China

^3^State Key Laboratory of Low Dimensional Quantum Physics and Department of Physics, Tsinghua University, Beijing 100084, China

^4^Hefei National Laboratory, Hefei 230088, China

^†^ Equally contributed to this work.

*Corresponding authors. E-mails: [zhuyy@baqis.ac.cn](mailto:zhuyy@baqis.ac.cn); [dingzhang@mail.tsinghua.edu.cn](mailto:dingzhang@mail.tsinghua.edu.cn); [qkxue@](mailto:qkxue@)mail.tsinghua.edu.cn

**Extended Data of Device A**

Figure S1 compares the $I$-$V$ characteristics at 1.6 K in two separate cool-downs. Similar to that observe at 5 K [Fig. 1(c), (d)], the critical Josephson current in the first cool-down is smaller than that in the fifth fool-down.

Figure S2(a) and (b) collect several representative $I$-$V$ characteristics of device A under microwave irradiations at 70 K—the temperature at which the previous report observed half-integer Shapiro steps [19]. In the first cool-down, apart from the main sequence of integer steps, we observe additional kinks at half integers. They can be better appreciated in the derivative traces (bottom panel), where small humps at $\pm1/2$, $\pm3/2$ and $\pm5/2$ exist between the sharp peaks at integers. By contrast, the same device shows only integer steps in the fifth cool-down, as illustrated by the curves in Fig. S2(b). The corresponding derivative curves now exhibit smooth arcs connecting the sharp peaks at integers of $hf/2e$.

Figure S3 shows $I$-$V$ characteristics of device A in three thermal cycles without or with the current training. In Figure S3a, we show the $I$-$V$ characteristics at $T =$ 10 K after one thermal cycle (from 120 K to 10 K) without applying any current during the cool-down, i.e., $I_{b}=0$ mA. The junction shows a single switch between the zero-bias branch and the normal state. The critical Josephson currents in the positive and negative directions are comparable, as can be further appreciated in Fig. S3(b) focusing on the zero-bias branch. The critical current is 350 μA. Figure S3(c) and 3(d) show the $I$-$V$ characteristics after another cool-down (from 120 K) with a training current of 1 mA. Interestingly, the $I$-$V$ characteristics now exhibit two transitions from the central branch to the normal state, as indicated by the red and black arrows. The zero-bias branch corresponds to a critical current of 84 μA, which is substantially smaller than that in the pristine state. In Fig. S3(e) and (f), we demonstrate that the original state—a single switch from the zero-bias branch to the normal state—is recovered after a third cool-down (from 120 K) without the current training.

In Figure S4, we present the color-coded plots of the Shapiro steps in device A after current annealing with $I_{b}=1$ mA. Half-integer Shapiro steps at $\pm5/2$ occur at 10 K. They vanish at higher temperature points. Similarly, we observe a clear half-integer Shapiro step at -3/2 after current annealing with $I_{b}=1.5$ mA in Fig. S5 at 10 K.

Figure S6 shows the evolution of the Shapiro steps as a function of in-plane magnetic field. Features at half-integers appear during the magnetic field sweeps. Furthermore, we observe indication of a fractional step at -1/4.

Figure S7 shows the critical current as a function of in-plane magnetic field without microwave. Typically, the standard Fraunhofer pattern should occur if the Josephson penetration depth is larger than the junction width. In our case, however, the Josephson penetration depth is shorter than the junction width [18]. Therefore, the experiment only shows a broad peak of $I_{c}$ as a function of $B_{x}$. The peak position shifts substantially due to the field sweeping. It centers around -0.7 mT in the up-sweep from -1.9 mT to 1.9 mT. By contrast, the peak position moves to about 0.75 mT in the down sweep from 1.9 mT to -1.9 mT. The remanent field of the electromagnet can be excluded as the origin of the hysteresis, because we determine the remanent field to be 0.04 mT after the field sweep by using the fluxgate magnetometer. Therefore, the hysteresis is intrinsic for the sample, indicating vortex trapping. The absence of the standard Fraunhofer pattern also indicates that the device can be treated as a wide junction, similar to that in refs. [10][28].


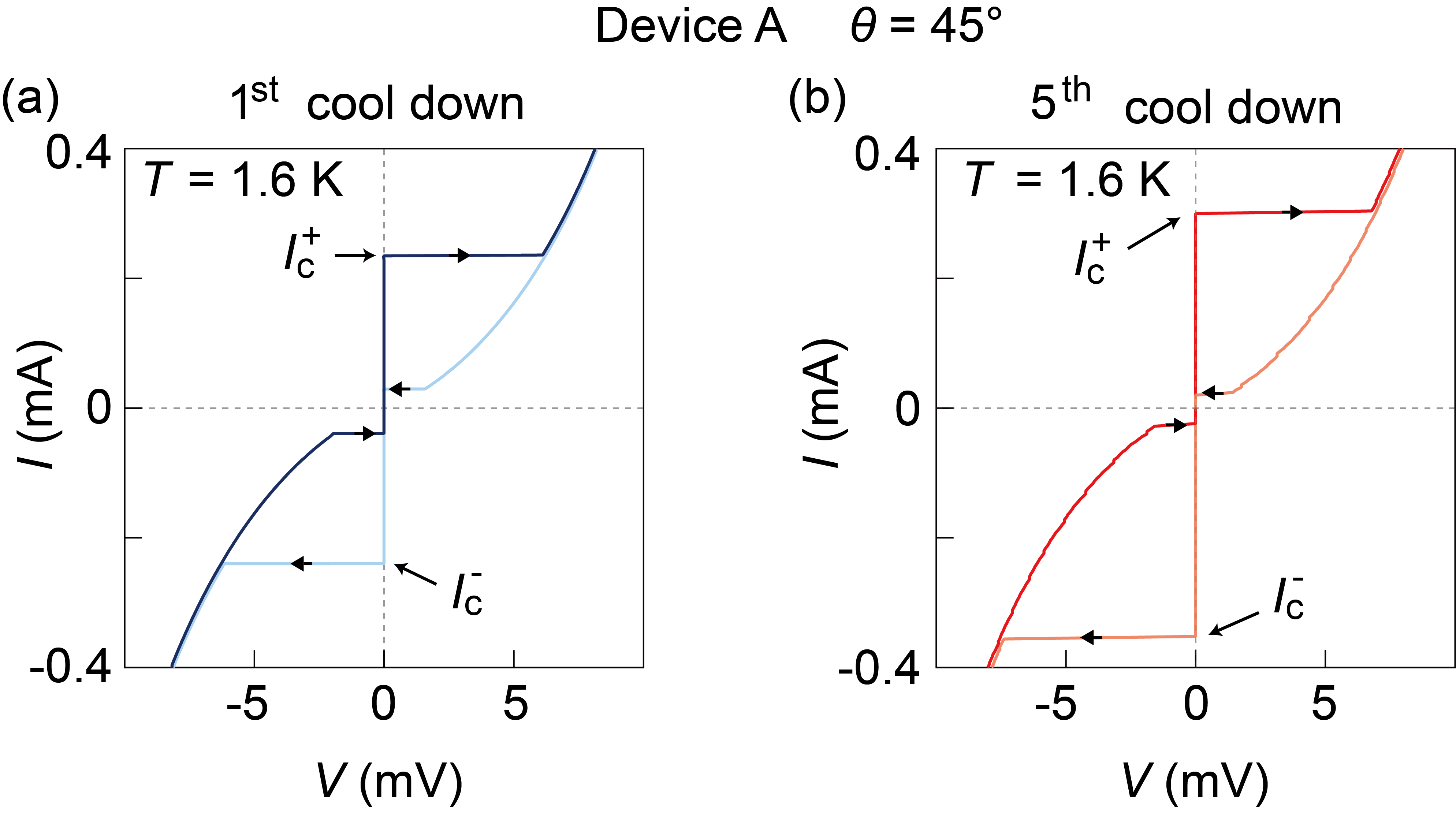


**FIG. S1:** (a), (b) $I$-$V$ characteristics in two separate cool-downs of device A. Arrows connecting the data indicate the sweeping directions. $I_{c}^{+}$ and $I_{c}^{-}$ mark the critical currents in the positive and negative directions.


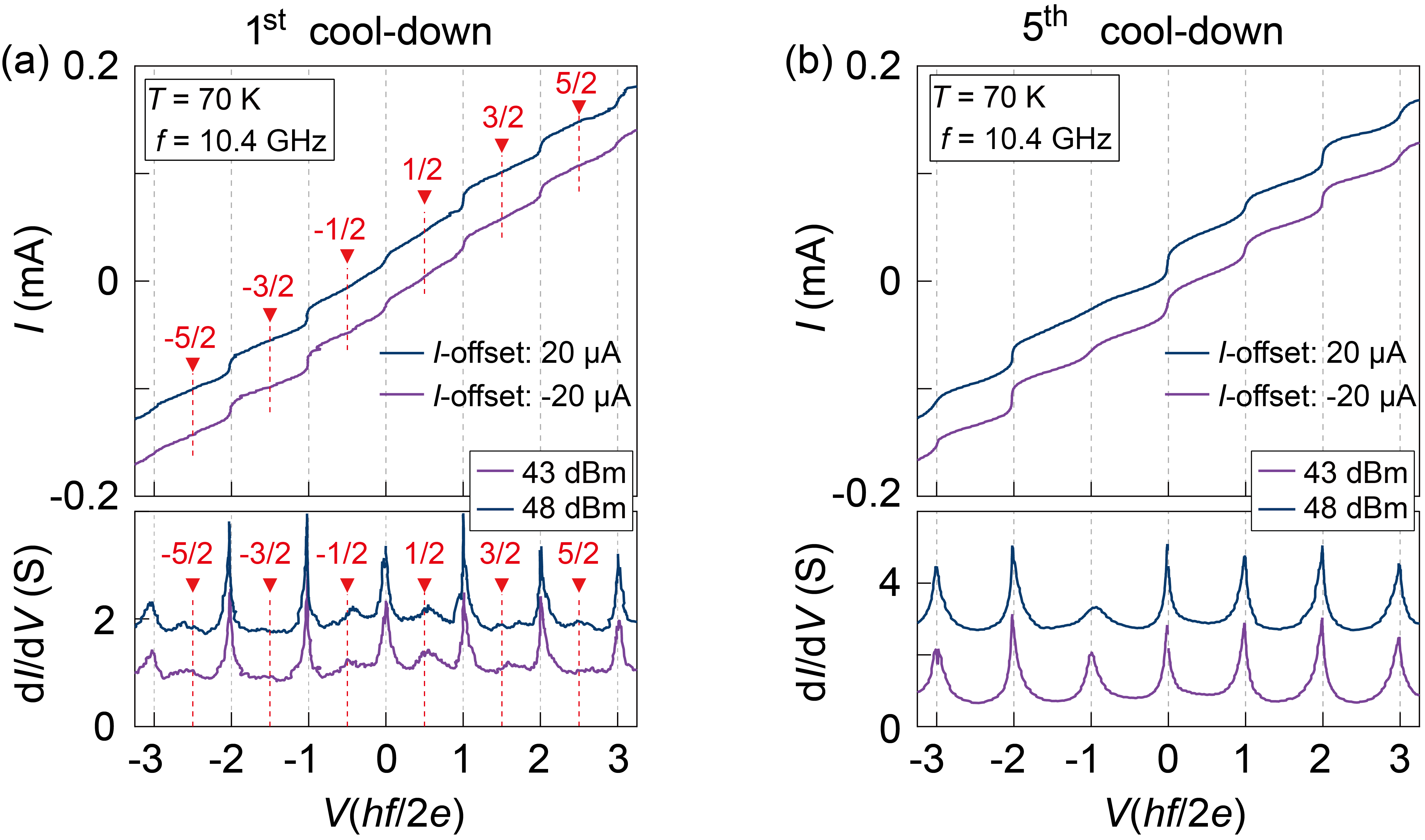


**FIG. S2:** (a), (b) Upper panels: Exemplary $I$-$V$ characteristics obtained in the two cool-downs under microwave irradiations with different powers. Curves are vertically offset for clarity. Lower panels: Tunneling conductance $dI/dV$ as a function of bias voltage obtained under the microwave irradiations. Curves are vertically offset for clarity.


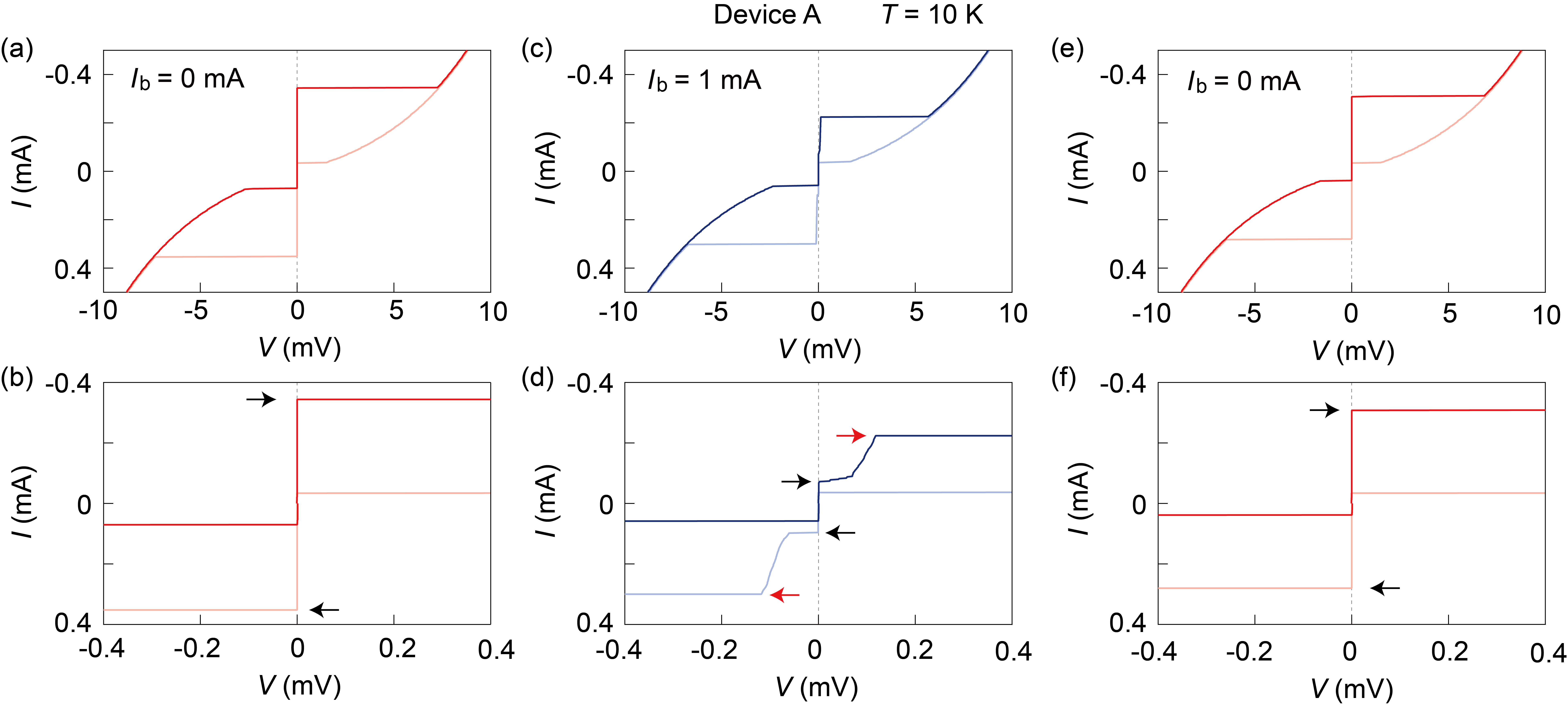


**FIG. S3:** $I$-$V$ characteristics in three cool-downs without or with the current training. (a) $I$-$V$ characteristics at 10 K with $I_{b}$ = 0 mA. (b) Zoom-in of the $I$-$V$ characteristics in (a) around zero bias. (c) $I$-$V$ characteristics at 10 K in a second cool-down with $I_{b}$ = 1 mA. (d) Zoom-in of the $I$-$V$ characteristics in **c** around zero bias. (e) $I$-$V$ characteristics at 10 K after a third thermal cycling with $I_{b}$ = 0 mA. (f) Zoom-in of the $I$-$V$ characteristics in (e) around zero bias. Black arrows mark the critical current at both positive and negative directions. Red arrows in (d) indicate the second switching from the zero-bias branch to the normal state.


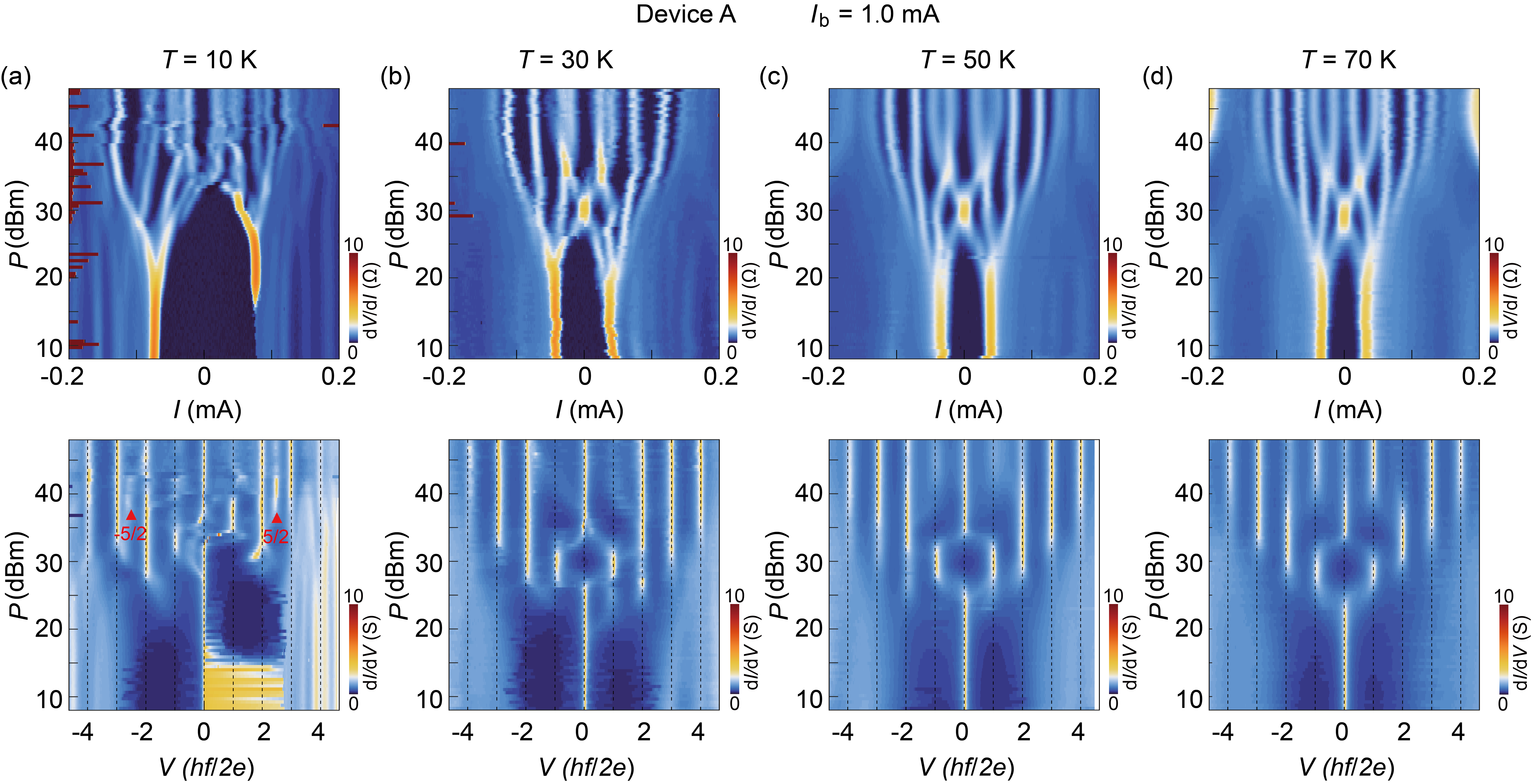


**FIG. S4:** (a)-(d) Color-coded plots of $dV/dI$ as a function of $P$ and $I$ (top panels) or $dI/dV$ as a function of $P$ and $V$ (in unit of ${hf}/{2e}$) for device A after current annealing with $I_{b}$ = 1 mA at four consecutively reached temperature points: 10 K, 30 K, 50 K, 70 K (in the warm-up direction). Dashed lines in the lower panels indicate the integer Shapiro steps. Red arrows mark the observed fractional Shapiro steps.


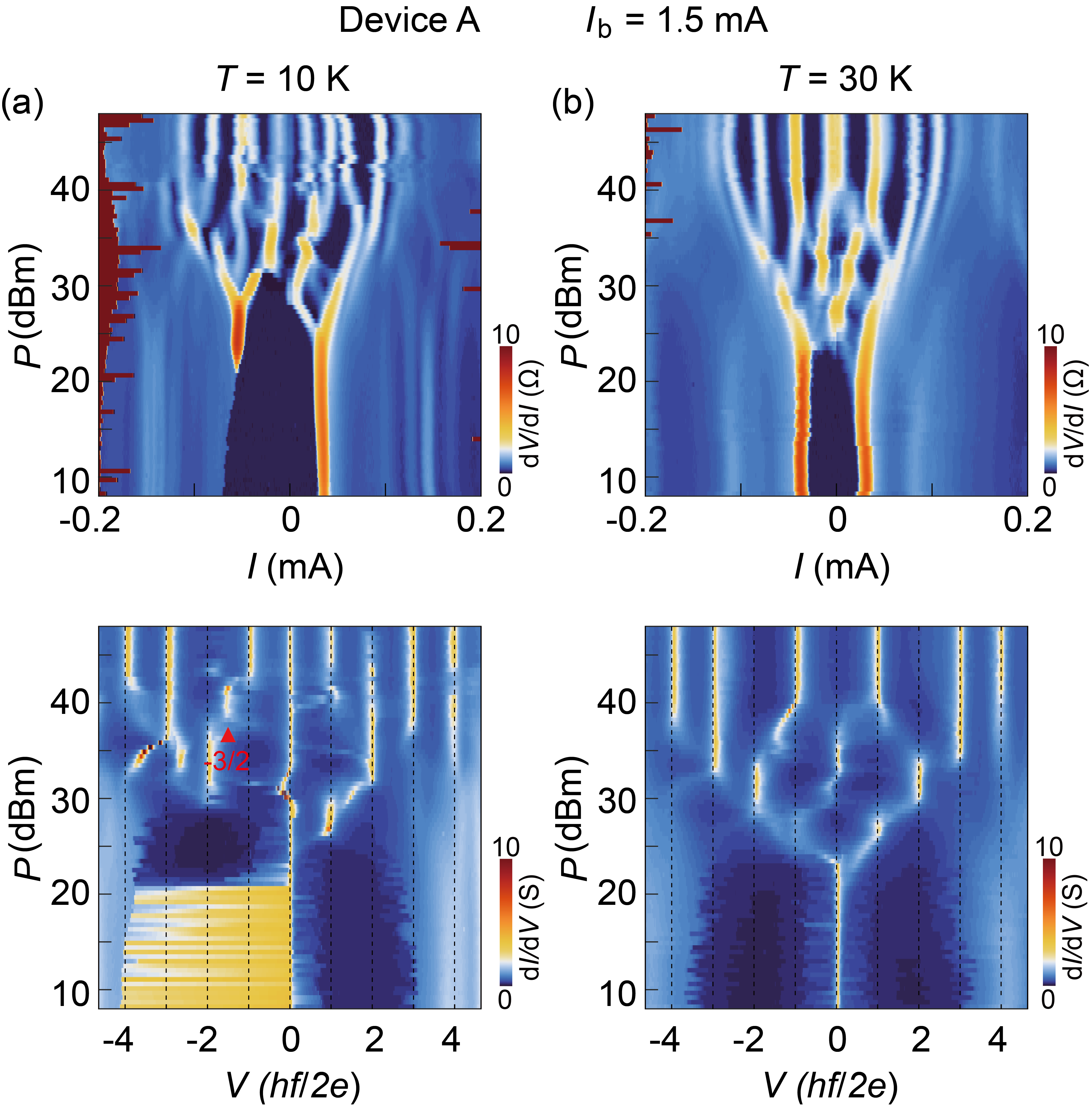


**FIG. S5:** (a), (b) Color-coded plots of $dV/dI$ as a function of $P$ and $I$ (top panels) or $dI/dV$ as a function of $P$ and $V$ (in unit of ${hf}/{2e}$) for device A after current annealing with $I_{b}$ = 1.5 mA at two consecutively reached temperature points: 10 K, 30 K (in the warm-up direction). Dashed lines in the lower panels indicate the integer Shapiro steps. Red arrow marks the observed fractional Shapiro step at -3/2.


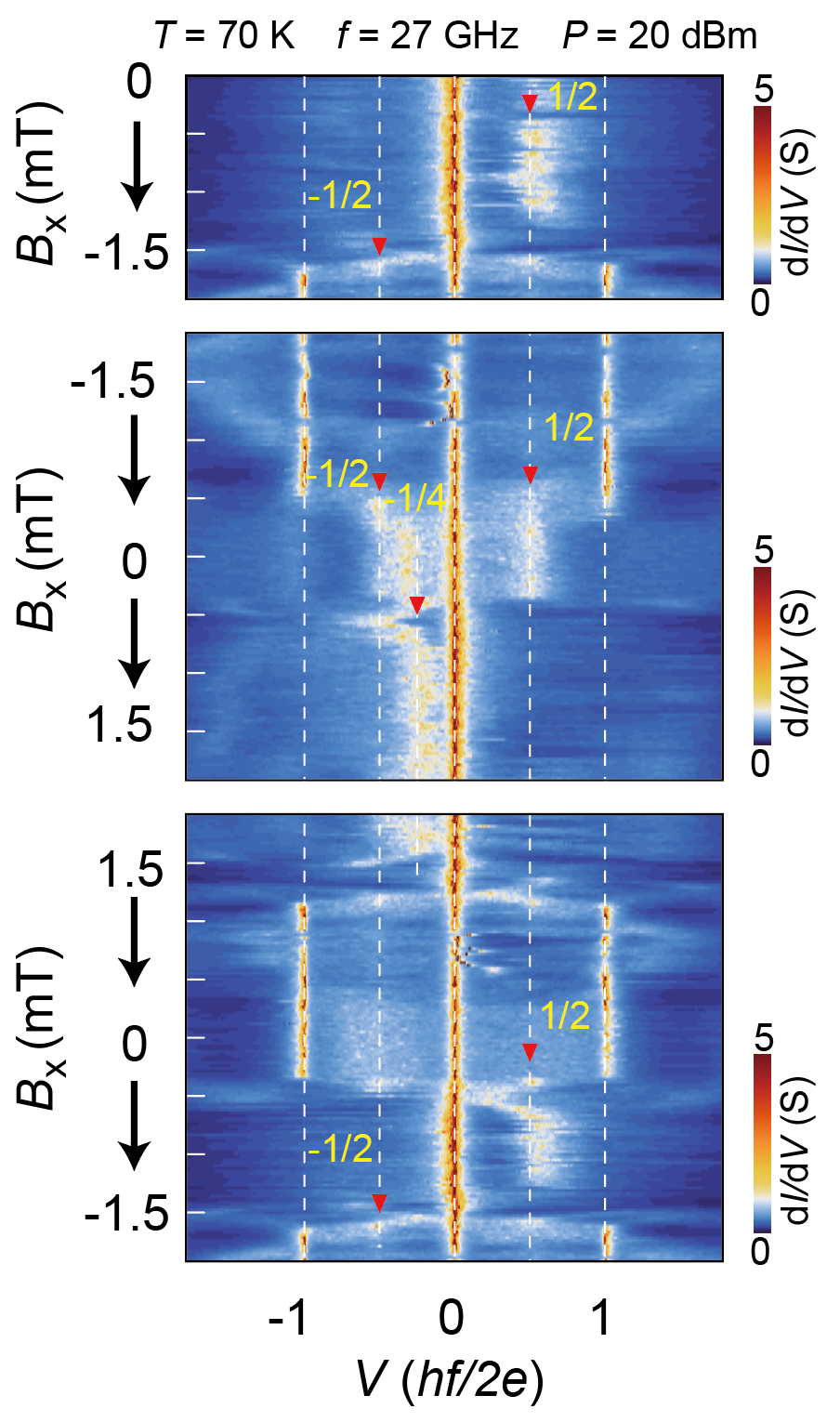


**FIG. S6:** $dI/dV$ as a function of $V$ and the in-plane magnetic field $B_{x}$ under microwave irradiations at 70 K. The microwave frequency is 27 GHz. The microwave power is 20 dBm. Black arrows on the left side indicate the sweeping directions of the magnetic field. Red arrows with numbers indicate even-denominator fractional Shapiro steps.


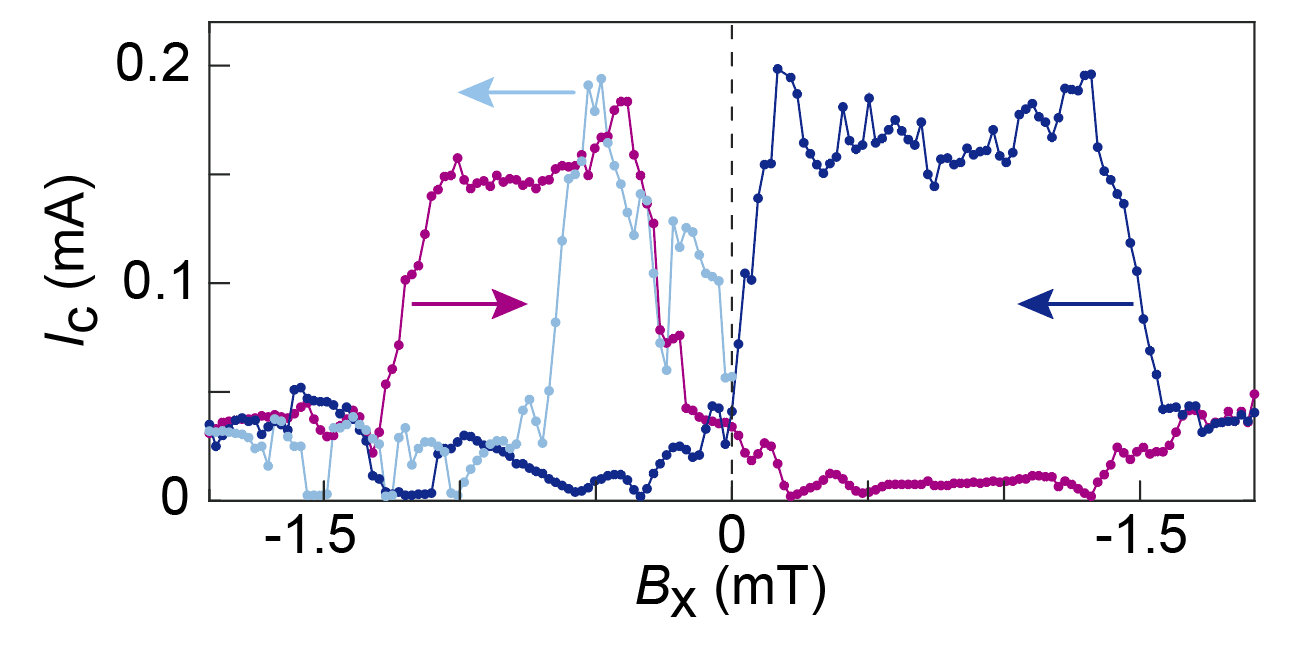


**FIG. S7:** Critical current $I_{c}$ as a function of in-plane magnetic field $B_{x}$ at 70 K for device A without microwave irradiation.

**Extended Data of Device B**

Figure S8 shows the temperature dependent junction resistance of device B, attesting to the high quality of the junction since its $T_{c}$ is the same as that of the bulk crystal.

Figure S9 presents the evolution of Shapiro steps at 10 K for device B. This measurement is carried out after current sweeps at intermediate temperature points at 70 K, 50 K and 30 K.

**
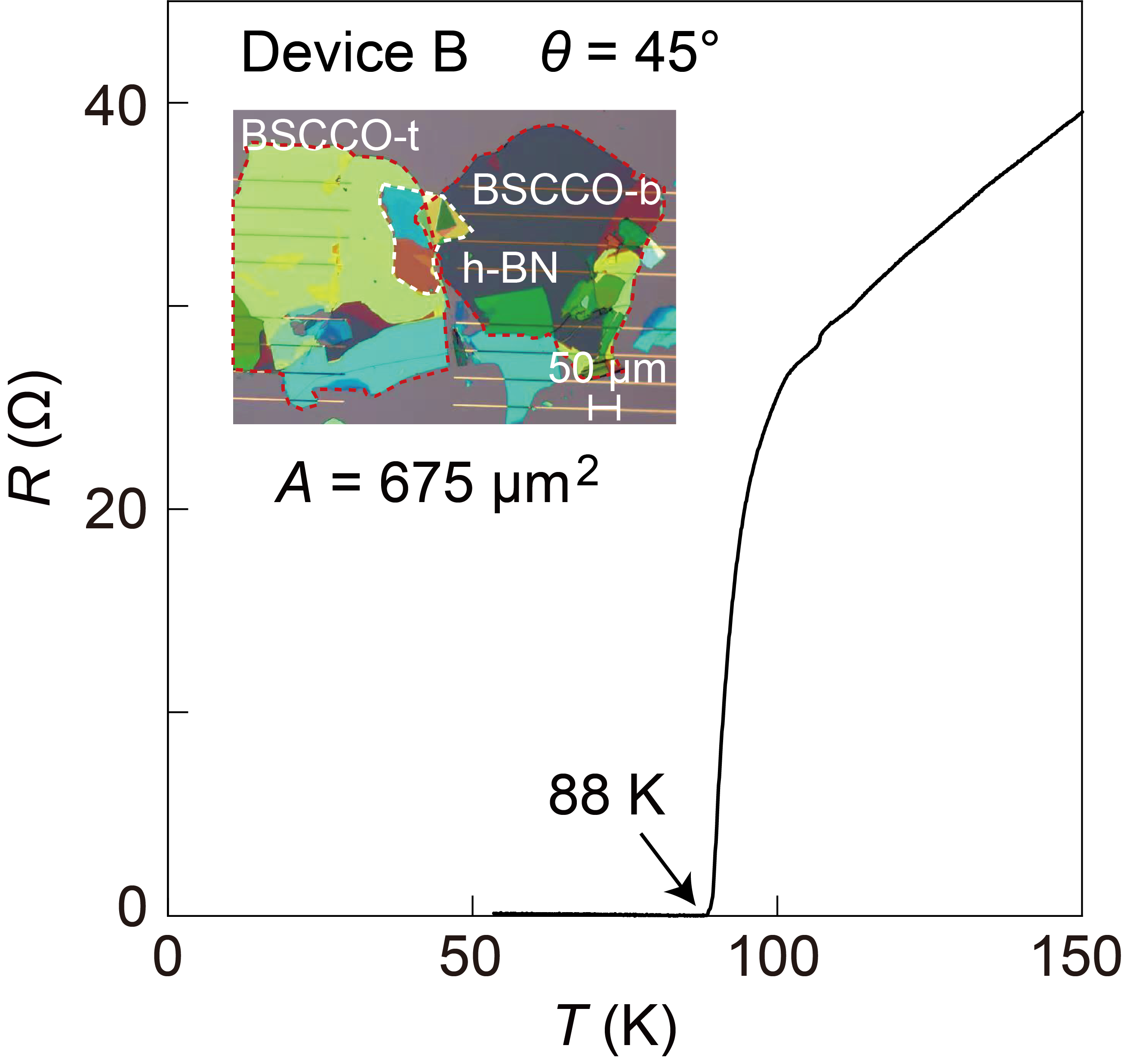
**

**FIG. S8:** Temperature dependent junction resistance of device B. Inset shows the optical image of device B. The top and bottom flakes of Bi_2_Sr_2_CaCu_2_O_8+x_ (BSCCO) are marked as BSCCO-t and BSCCO-b, respectively.


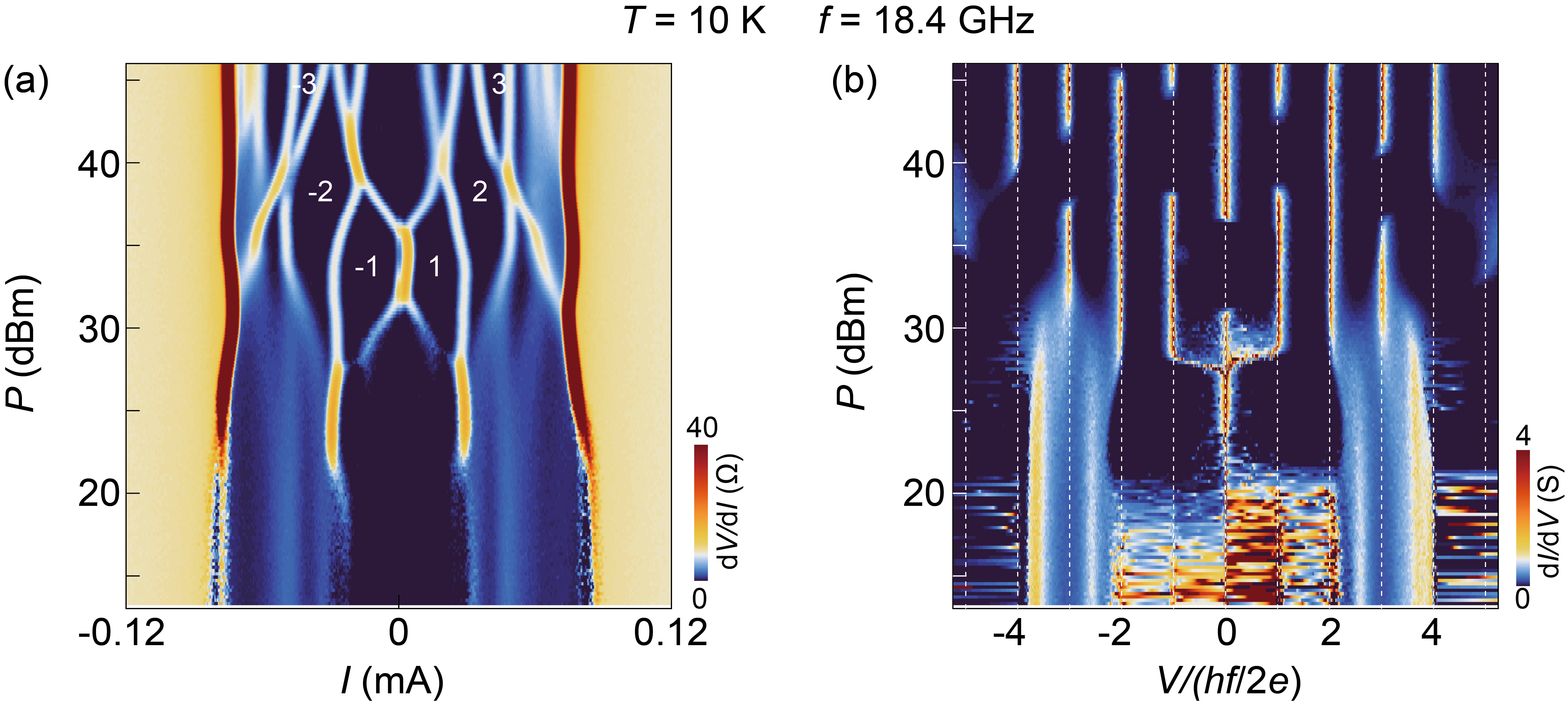


**FIG. S9:** (a) Color coded plot of $dV/dI$ as a function of $P$ and $I$ at 10 K (in the same cool-down as that for Fig. 2f-k in the main text). Numbers indicate the sequence of Shapiro steps. (b) $dI/dV$ as a function of $P$ and $V$. Dashed lines indicate the positions of integer Shapiro steps.

**Extended Data of Device E**

Figure S10(a) shows the dc Josephson effect of device E without microwave irradiation. Shining microwave on this device in the initial cool-down causes suppression of the critical current, as demonstrated in Fig. S10(b). Well-defined Shapiro steps are missing in this sample, before applying any current training or magnetic field. Figure S10(c) shows that half-integer Shapiro steps appear after sending the current pulses to the sample at 30 K.


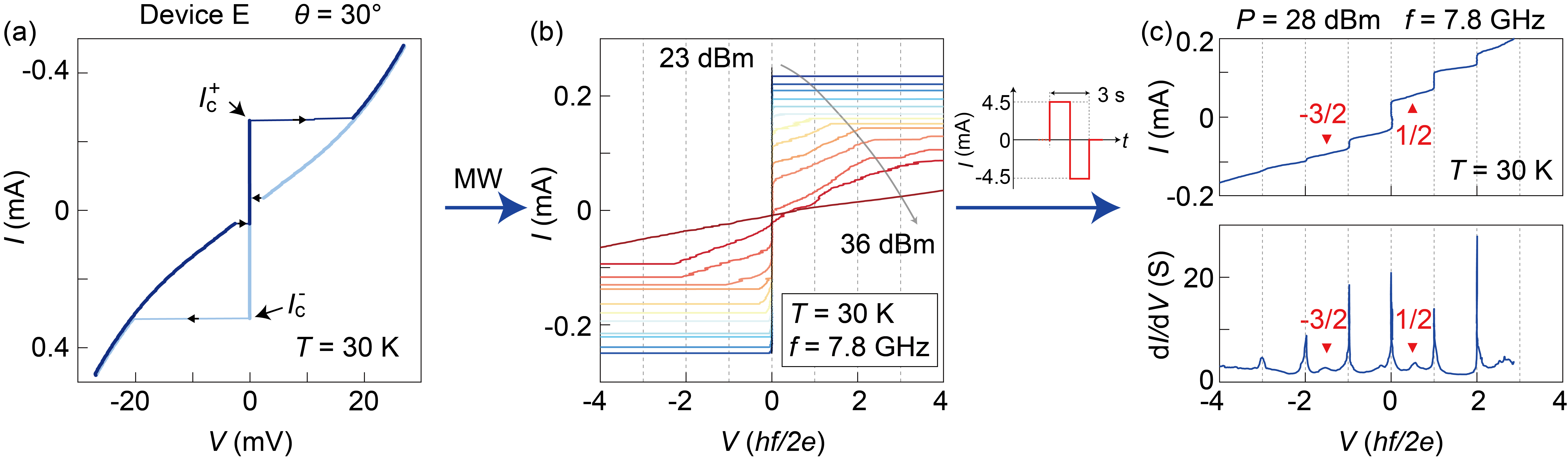


**FIG. S10:** (a) $I$-$V$ characteristics for device E with a twist angle of 30° at 30 K. (b) $I$-$V$characteristics of device E under microwave irradiations. Microwave power ranges from 23 dBm to 36 dBm (in step of 1 dBm). (c) $I$-$V$ characteristics and corresponding $dI/dV$ of device E under microwave irradiation after applying the current pulses [typical wave form shown between panel (b) and (c)]. Arrows indicate the half-integer Shapiro steps.
